# Vineyard Design, Cultural Practices and Physical Methods for Controlling Grapevine Pests and Disease Vectors in Europe: A Review

**DOI:** 10.3390/insects17010113

**Published:** 2026-01-20

**Authors:** Francesco Pavan, Elena Cargnus, Pietro Zandigiacomo

**Affiliations:** Department of Agricultural, Food, Environmental and Animal Sciences (DI4A), University of Udine, Via delle Scienze 206, 31000 Udine, Italy; elena.cargnus@uniud.it (E.C.); pietro.zandigiacomo@uniud.it (P.Z.)

**Keywords:** cultural control, physical control, habitat management, agronomic practices, flavescence dorée, bois noir

## Abstract

In Europe, due to reduced availability and efficacy of active ingredients, strategies against grapevine pests based on alternative tools to synthetic pesticides need to be developed. So far, attention has been mainly focused on biological control (arthropod natural enemies and entomopathogens) and mating disruption, but other means can also help keep pests below economic injury levels. This paper aims to review information on the direct effects of farmers’ choices on grapevine pest populations, ranging from vineyard design (e.g., growing habitat, grapevine cultivar, and training system) to annual agronomic practices (e.g., fertilization, irrigation, and pruning), and specific cultural and physical methods. Information was based on the CABI Digital Library, websites and books on grapevine pests. The data presentation is based on control strategies rather than pests, as it was considered more important to focus on the mode of action of different practices and to know which pests they affect simultaneously. The widespread availability of insecticides has long led to the neglect of the potential of cultural practices, which can effectively integrate other pest control tools.

## Simple Summary

In Europe, grapevine pest control requires alternative tools to synthetic insecticides due to the withdrawal of many active ingredients and the development of pest resistance. Against certain pests, biological control and mating disruption can be effectively used. However, the probability that pests exceed the economic injury levels also depends on decisions made when planting the vineyard and on the agronomic practices adopted each year. Moreover, specific cultural and physical methods may be adopted to control certain pests. This paper aims to review information on these tools, which, neglected for many years as unnecessary, can represent eco-friendly alternatives to insecticides for controlling grapevine pests.

## 1. Introduction

In European viticulture, the development of Integrated Pest Management (IPM) strategies against grapevine pests and disease vectors is a desirable alternative to the exclusive use of synthetic pesticides to reduce negative effects on the environment and human health. Still, it is also becoming mandatory due to the reduced availability of insecticides and acaricides, resulting from the withdrawal of many active ingredients and the development of pest resistance.

Among alternative control strategies to synthetic insecticides and acaricides, the most widely used and effective in viticulture are biological control and mating disruption. The most successful examples of biological control in vineyards include (i) the release of phytoseiid mites (Acari: Phytoseiidae) resistant to certain pesticides to achieve long-term control of tetranychid mites (Acari: Tetranychidae) [1,2,3], (ii) the sprayings of *Bacillus thuringiensis* Berliner against grapevine moths [*Lobesia botrana* (Denis & Schiffermüller) and *Eupoecilia ambiguella* (Hübner) (Lepidoptera: Tortricidae)] [4,5,6], and (iii) the periodic release of the coccinellid predator *Cryptolaemus montrouzieri* Mulsant (Coleoptera: Coccinellidae) and the hymenopteran parassitoid *Anagyrus vladimiri* (Girault)] (Hymenoptera: Encyrtidae) against *Planococcus ficus* (Signoret) (Hemiptera: Pseudococcidae) [7,8,9,10]. Mating disruption has proven decisive in controlling grapevine moths [11,12,13] and makes a valuable contribution to maintaining *P. ficus* at non-harmful densities [9,14,15,16]. Natural products, such as pyrethrin, spinosad, insecticidal soap, and orange oil, can sometimes replace synthetic insecticides, although their efficacy is often lower [5,9,17,18,19].

In general, agronomic choices and the management of surrounding habitats can influence pest infestations and damage [20,21]. This review aims to summarize current knowledge on the direct effects of farmers’ choices on grapevine pest populations, ranging from vineyard design (e.g., growing habitat, grapevine cultivar, and training system) to annual agronomic practices (e.g., fertilization, irrigation, and pruning), and specific cultural and physical methods. Grapevine cultivar choice will be considered only when it is a decisive factor. Natural enemies will be mentioned only when a practice adopted against a pest can also interfere with the biological control of one or more pests.

## 2. Materials and Methods

Bibliographic research on strategies that reduce grapevine pest (or disease vector) populations or their damage through methods other than chemical, biological and semiochemical control was conducted using the CABI Digital Library. Classic and recent books on grapevine pests, as well as websites, were also consulted.

The collected information was organised according to the scheme reported in Table 1. The presentation scheme was partially suggested by those used in IPM manuals [20,21] and a review [22]. A presentation by control strategy was preferred over a pest-by-pest approach, as focusing on the mode of action of each strategy can stimulate its improvement and provide the conceptual basis for developing it against other pests. Furthermore, this structure allows us to know if a practice simultaneously affects multiple pests. However, to provide concise information, the strategies listed in Table 1 have been referenced to each pest in Table 2.

Strategies to control vectors of phytoplasma associated with Grapevine Yellows Diseases (GYDs) will be presented in a separate section.

Information on the biology and damage caused by each species is normally reported only at its first mention. However, a summary table outlining the biology, symptoms and damage of the pests considered is provided (Table S1 in Supplementary Materials).

## 3. Selection of the Vineyard Planting Site

The site of grapevine planting can influence arthropod pest populations and the extent of their damage, depending on climatic conditions, soil characteristics, and other habitat components such as altitude, slope, orientation and landscape diversity.

### 3.1. Climatic Conditions

The average annual temperature is a key factor that determines the number of generations a pest species can complete each year [23]. In cooler grape-growing regions, multivoltine species complete fewer generations per year, often resulting in reduced damage risk and insecticide applications. In this context, climate warming has increased the number of generations and expanded the geographical distribution of some pests [24,25], making them more harmful where they were already present and have become so in regions where they were previously absent.

The green leafhopper *Hebata vitis* (Göthe) (Hemiptera: Cicadellidae) is a polyphagous phloem feeder on grapevine leaf veins, causing reddening or yellowing of leaf margins followed by desiccation [26,27,28,29]. Leaf symptoms are associated with reduced photosynthesis and transpiration rates [30], which can lead to yield losses and a reduction in sugar content in grape berries [31,32,33]. The leafhopper overwinters as adults outside vineyards and usually completes two to three generations per year [26,27,34,35]. However, in a grape-growing area where the leafhopper usually has three generations, a fourth generation was observed in the warmest years [36]. In grape-growing regions with low rainfall, water stress occurring after the peak of *H. vitis* nymphs amplifies symptom expression, and consequently, the likelihood of economic damage [32].

The green leafhopper *Jacobiasca lybica* (Bergevin & Zanon) (Hemiptera: Cicadellidae), which causes leaf symptoms similar to those of *H. vitis*, is expanding its distribution due to climate warming. For example, in Italy, the leafhopper, previously reported only for Sardinia and Sicily [37,38,39], has recently become harmful also in the Apulia region [40].

The European fruit scale, *Parthenolecanium corni* (Bouché) (Hemiptera: Coccidae), is a polyphagous species that can cause severe damage to grapevines [41]. It is a phloem feeder that overwinters as second-instar nymphs under bark and can complete one or two generations per year. The occurrence of a second generation was first observed in southern Italy [41] and subsequently in northern Italy [42]. In spring and summer, nymphs and females feed on leaves, shoot axes and bunches, causing both qualitative and quantitative damage, mostly associated with honeydew excretion and the development of sooty mold. As long as it completed only one generation per year, it was considered to be of little harm [43], while serious damage was associated with completing two generations [41,42]. Once again, the role of climate warming in making a species more harmful emerges.

The larvae of grape berry moths, *L. botrana* and *E. ambiguella*, are anthophagous during the first generation and carpophagous during the summer generations [44,45,46,47]. Their damage consists of yield and quality losses, the latter associated with the spread of bunch rots facilitated by larval erosion [48,49,50,51,52]. *Lobesia botrana* completes two to four generations per year, whereas *E. ambiguella* produces two to three [44,45,47,53]. *Eupoecilia ambiguella* is restricted to and harmful only in cooler grape-growing regions (northern ones), whereas *L. botrana* prefers warmer grape-growing regions (southern ones) with transitional ones, where both species coexist [45,53]. For *L. botrana*, climate warming determined an increase in the number of generations per year that the species develops across different grape-growing regions [54,55,56,57,58,59,60,61,62], and a shift toward higher altitude vineyards has also been suggested [59]. Since prolonged wetness favors the spread of *Botrytis cinerea* Person: Fries [63], the qualitative damage caused by grape berry moths associated with the fungus can be reduced by growing grapevines in regions with low rainfall and good ventilation, facilitating the quick drying of the bunches.

The vine bud moth, *Theresimima ampellophaga* (Bayle-Barelle) (Lepidoptera: Zygaenidae), is a univoltine species in northern regions and bivoltine in southern ones, overwintering as young larvae on woody parts, and completes the entire life cycle on *Vitis* species, feeding on buds before sprouting and on leaves in spring and summer [44,64,65,66,67,68]. Damage from *T. ampellophaga* is more frequent in hilly vineyards bordering groves, as the species prefers warm and slightly humid habitats [66,67,69]. In summer, storms characterized by heavy rain and wind cause older larvae to fall [65]; therefore, an increased frequency of such events could be detrimental to the species [68]. Climate warming could instead make the species bivoltine even in the northernmost distribution areas.

The tetranychid mite *Eotetranychus carpini* (Oudemans) (Acari: Tetranychidae) feeds on the leaf mesophyll, causing chromatic alterations localized especially along the veins (i.e., yellowing in white cultivars and reddening in red cultivars), which may be followed by drying and premature leaf drop [70]. The mite is more widespread in hilly and sunny vineyards [71] because it prefers moderate temperatures and low relative humidity [1].

### 3.2. Soil Characteristics

The grape phylloxera, *Daktulosphaira vitifoliae* (Fitch) (Hemiptera: Phylloxeridae), is a species native to North America, where it induces the formation of leaf and root galls on American *Vitis* species [72]. Following its introduction into Europe, around the 1860s, it destroyed the vineyards of *Vitis vinifera* Linnaeus, as root damage associated with the radicicole activity caused the collapse of the root system [44,73]. However, grapevines grown in sandy soils are less affected by *D. vitifoliae* as the radicicoles are unable to disperse through the loose sandy texture, limiting the spread from infested to uninfested roots [44,72]. In clay soils subject to water stagnation, the development of phylloxera is also discouraged [72]. Soil characteristics such as low aluminium exchange capacity and acidic pH are associated with higher grape phylloxera abundance [74].

The scarab beetle *Anomala vitis* Fabricius (Coleoptera: Scarabaeidae) is a polyphagous and univoltine pest whose adults feed on broadleaf trees and can occasionally cause defoliation in young vineyards [44,67,75]. Adults feeding on grapevine leaves derive from larvae that have developed on the roots of herbaceous plants. Since the larvae prefer sandy soils for development, damage to grapevines is more frequent when vineyards are planted on this type of soil.

Larvae of other scarab beetles, [e.g., *Melolontha melolontha* (Linnaeus) (Coleoptera: Scarabaeidae) and *Pentodon bidens punctatus* (Villers) (Coleoptera: Scarabaeidae)] can erode the roots and collar of newly planted grapevines, even causing plant death [44,66,67,76]. Damage caused by *P. bidens* is more frequent in vineyards planted on soils previously occupied by permanent meadows [43].

Soil fertility, which promotes grapevine vigor, may also be associated with heavy infestations by sap-feeding insects and damage caused by grape berry moths [see Section 4.2.1 for the effects of nitrogen fertilizing and irrigation on the population density of *Panonychus ulmi* Koch (Acari: Tetranychidae), *H. vitis* and *P. ficus,* and damage of grape berry moths].

### 3.3. Other Habitat Components

Besides climatic conditions and soil characteristics, vineyard position (plane or hilly), slope exposure (north- or south-facing) and the features of the surrounding habitats can all influence the abundance of pests in vineyards.

Based on the *B. cinerea* biology, fungus damage associated with *L. botrana* and *E. ambiguella* should be lower in hilly vineyards, especially those south-facing, which exhibit reduced vine vigor and shorter wetness duration (for the mode of action and references, see Section 3.1 and Section 4.2.1).

The honeydew moth *Cryptoblabes gnidiella* (Millière) (Lepidoptera: Pyralidae) is a berry moth that overwinters in the larval stage into dried bunches and in Italy and France completes four generations per year [77,78]. The larvae begin feeding on the green tissues of the cluster, rachis, and pedicels, reducing the sap flow to the berries, which tend to wither, and then also on the berries themselves. Infestations are most frequent in the littoral areas of Italy and France [78,79,80].

At grapevine bud break, larvae of noctuid moths belonging to the genus *Noctua* [e.g., *N. pronuba* (Linnaeus) and *N. fimbriata* (Schreber)] (Lepidoptera: Noctuidae) can erode buds and shoots [44,66,67,81]. Infestations are more frequent in terraced vineyards, those surrounded by woods, or those bordering slopes [82].

The spotted wing fly, *Drosophila suzukii* (Matsumura) (Diptera: Drosophilidae), is a highly polyphagous and multivoltine species native to East Asia that causes severe economic losses on soft fruits and cherries [83], and can also lay eggs on grapes of cultivars with soft skin [84,85,86,87,88,89,90]. Larger populations were observed in hilly vineyards compared to those in the plains, likely due to the greater presence of both overwintering and alternative host plants (see Section 5.1) [85,91].

## 4. Host-Plant-Mediated Pest Control

Grapevine species or cultivars, as well as the agronomic practices adopted to grow them, can influence susceptibility and sensitivity to arthropod pests. More susceptible *Vitis* species or cultivars exhibit higher infestation levels than others when grown under the same environmental conditions. More sensitive *Vitis* species or varieties suffer greater injury levels than others when exposed to the same infestation levels and environmental conditions. However, the adopted agronomic practices can either amplify or minimize both susceptibility and sensitivity in a given cultivar.

### 4.1. Choosing Vitis Species or Cultivar to Plant

The tetranychid mite *E. carpini* shows a preference for cultivars with a pubescent leaf undersurface [1].

Grapevine cultivars showed different degrees of susceptibility and sensitivity to *H. vitis* [44,92]; however, the susceptibility can also be mediated by the influence that the cultivar has on the activity of the leafhopper egg parasitoid *Anagrus atomus* (Linnaeus) (Hymenoptera: Mymaridae) [93].

The roots of American *Vitis* species and their hybrids are not severely damaged by the feeding activity of *D. vitifoliae* radicicoles [72]. Grafting *V. vinifera* onto American rootstocks saved European viticulture from phylloxera.

The vine mealybug *P. ficus* is a polyphagous and multivoltine species that causes severe damage in vineyards worldwide. It is a phloem feeder that mostly overwinters as fertilized females and develops by feeding on woody parts, living under the bark, on leaves and within bunches [7,94,95]. Mealybugs can adversely affect yield and fruit quality by infesting woody parts of the grapevine and fouling leaves and bunches with honeydew excretion, which promotes the development of sooty mold. Grapevine cultivars showed different degrees of susceptibility to *P. ficus* [96]. Moreover, bunches of late-harvested cultivars are more heavily damaged by *P. ficus* due to prolonged exposure to later mealybug broods [7]. Cultivar susceptibility must also be assessed in relation to Grapevine leafroll-associated virus 3 (GLRaV-3), causing the grapevine leafroll disease (GLD), for which *P. ficus* is one of the main vectors [97,98]. This disease reduces yield and above all sugar content in berries, making it necessary to remove heavily infected vineyards [99]. To prevent the spread of GLRaV-3, virus-infected grapevines must be promptly identified and insect vectors controlled. For this reason, even a cultivar that is poorly susceptible to *P. ficus* must be considered highly susceptible if it is so to the virus itself, especially considering that fast disease spread can occur even at low mealybug population densities [100].

The grapevine cultivar influences susceptibility to the first generation of *L. botrana* and *E. ambiguella*, with infestation levels positively correlated with inflorescence earliness and negatively correlated with inflorescence hairiness [44,101,102]. However, the first generation can cause yield losses only on cultivars with lower fruit setting [48]. The susceptibility to carpophagous generations of the two berry moths is also influenced by cultivars [44,46,103,104,105]; in the case of *L. botrana*, it is associated with egg-laying preferences and differences in larval settlement and survival [105,106,107,108,109], with the implication of morphological and chemical traits of the cultivar [44,103,104,110]. Females of *L. botrana* dislike laying eggs of the last annual generation on cultivars close to harvest [54,104,111], so this generation preferentially infests late-harvested cultivars [55,56]. Cultivar also influences the sensitivity to the second-generation of *L. botrana*, since (i) yield losses are lower in late-harvested cultivars, because healthy berries partially compensate for the weight loss of damaged ones [112,113], and (ii) the spread of *B. cinerea* to berries adjacent to those bored by larvae is reduced [114].

As the captures of the *C. gnidiella* adults substantially increase from the beginning of August onwards, the early-harvested cultivars are much less infested than the late ones [78].

Economic damage by *Noctua* spp. can occur on early-sprouting cultivars because shoot erosion results in the regrowth of sterile shoots [81].

*Drosophila suzukii* prefers grapevine cultivars with soft-skinned berries [85,88,90,115].

Although it is not a decisive factor in cultivar choice, knowledge of cultivar susceptibility to pests is useful for decision-making in the IPM context. For grape berry moths and *H. vitis*, the relative sensitivity of cultivars enables the differentiation of economic injury levels and action thresholds [32,49,113]. Moreover, in multi-cultivar vineyards that are homogeneous for grapevine age, training system and agronomic practices, the sampling times for action threshold exceeding can be reduced following this sequential procedure: (i) the most susceptible cultivars should be sampled first; (ii) the least susceptible cultivars, considering their decreasing relative infestation level risk, should be sampled only when the action threshold is exceeded in the more susceptible ones; and (iii) sampling can be stopped when the action threshold is not exceeded in cultivars with higher relative infestation levels.

### 4.2. How to Grow Grapevines

Agronomic practices, such as fertilizing and irrigation, training systems and pruning, can favor sap-feeding pests by (i) increasing body size and fecundity; (ii) providing better shelter from high temperatures and low relative humidity; and (iii) reducing foliar insecticide coverage. Soil management and harvest timing can also influence the extent of pest damage.

#### 4.2.1. Fertilization and Irrigation

The European red mite *P. ulmi* overwinters as eggs on canes and two-year-old branches and completes many generations per year by feeding on leaf mesophyll [43,44,116,117]. In spring, it can inhibit shoot development, affecting the availability of fruiting arms during the subsequent winter pruning. In summer, symptoms such as discolored leaf spots that progress to bronzing, drying and premature leaf drop, and the resulting loss of leaf functionality can reduce yield and fruit quality [118]. Nitrogen fertilization favors the growth of the mite population [44,119]. Surface irrigation increases infestation levels compared to non-irrigated control [120].

Infestation levels of *H. vitis* are favored by higher plant vigor [121,122], which increases the number of eggs laid per leaf by females [93]. Irrigated vineyards can exhibit higher infestation levels than non-irrigated ones [123] (for details, see Section 6.2); however, water stress following *H. vitis* infestation increases symptom expression and damage caused by the leafhopper [32]. Moreover, drip irrigation has been associated with higher pest population levels than sub-irrigation, because it creates a moist microclimate that is more favorable to adult colonization [123].

While annual applications of composted green waste for three consecutive growing seasons promoted *D. vitifoliae*, composted winery waste significantly reduced it [74].

Females of *P. ficus* exhibited higher survival and fecundity, larger body size and shorter development time on grapevines with higher nitrogen fertilization rates [124]. It suggests the importance of implementing balanced fertilization plans within IPM programs to reduce population densities and prevent pest outbreaks. The use of cover crops, which reduce nitrogen availability to grapevines, represents an effective strategy for lowering *P. ficus* infestations in vineyards [125].

Damage by *P. corni* mostly occurs in vineyards subjected to nitrogen fertilization and irrigation [41].

Lower infestation levels by *L. botrana* were associated with lower nitrogen fertilization [126]. Since bunch-rot damage is favored by high plant vigor [63,127], nitrogen fertilization and irrigation increase the grape berry moths’ damage component associated with the spread of *B. cinerea*.

Since the presence of solid organic material in the soil near young grapevines attracts *P. bidens punctatus* larvae, when establishing new vineyards in succession to meadowlands, it is advisable to avoid planting grapevine grafts rooted in peat substrates, fertilizing with manure and organically mulching (e.g., with pine bark) [66].

#### 4.2.2. Training System and Pruning

The grape erineum mite *Colomerus vitis* (Pagenstecher) (Acari: Eriophyidae) overwinters as a female under the outer bud scale and produces several generations per year, feeding on the leaves of the grapevine, on which felty patches (‘erinea’) appear [70,128]. The yield and quality of canes suitable for fruit arms can be reduced. Moreover, in Europe, it is the vector of the grapevine Pinot gris virus (GPGV) [129]. The spring infestation of *C. vitis* is higher in training systems with long canes (e.g., Guyot) than in those with short canes (e.g., spur-pruned cordon), because the overwintering populations prefer the middle and distal parts of the lignified shoots [130].

Bunch-zone leaf removal reduced *H. vitis* population in one study [131], likely due to a decrease in grapevine canopy density, while it had no effect in another [132].

The first generation of *P. ficus* develops mainly under the grapevine bark, while a significant portion of second-generation crawlers migrates to leaves and bunches [94,95]. Bunch colonization by second-generation crawlers is reduced in training systems where the bunches hang away from the trunk and cordons [7]. Therefore, defoliation and thinning of bunches in direct contact with the trunk and permanent cordons discourage colonization of leaves and bunches by second-generation *P. ficus* crawlers [133].

Grape berry moths’ infestations are higher in horizontal training systems (e.g., Pergola) than in vertical training systems, as the former favor egg laying and larval survival [134,135]. Bunch-zone leaf removal reduces *L. botrana* larval infestation (for details, see Section 6.2) and the development of *B. cinerea* associated with larval activity [126,136]. The training system and spring-summer pruning, which influence canopy structure, can affect *L. botrana* infestations by interfering with insecticide coverage of the bunches [137].

#### 4.2.3. Harvest Time

Since late-harvested cultivars are more susceptible to *P. ficus* damage [7], delaying harvest is likely to increase mealybug damage.

At bunch ripening, damage caused by *L. botrana*, *E. ambiguella* and *C. gnidiella* increases rapidly, as both the number of berries bored per active larva and the number of rotten berries rise [78,114,138]. For this reason, in the presence of *L. botrana* and *C. gnidiella* infestation, harvest must not be delayed beyond what is strictly necessary.

## 5. Interference with Vineyard and Grapevine Colonization by Pests

This section focuses on cultural control strategies aimed at reducing vineyard and grapevine colonization by (i) removal of pests’ source plants; (ii) physical exclusion of pests; and (iii) diverting pests from grapevines.

### 5.1. Removal of Pests’ Source Plants

The sources of pests towards grapevines can be represented by (i) alternative host plants (grapevine is one of the plant species on which a pest can complete its development); (ii) host plants of pests of which grapevines are only an occasionally feeding host (i.e., pest cannot complete its development on grapevine); (iii) overwintering host plants for adults (grapevine is the reproductive host, but adults overwinter feeding on other plant species); and (iv) summer host plants for adults (grapevine is the reproductive host, but adults migrate during summer to feed on other plant species). Although it is not always possible to remove external plants that act as sources of pests, this knowledge allows us to identify vineyard plots at risk and to focus sampling and control measures only along edges adjacent to these potential sources, as suggested by decreasing gradients of infestation from these edges of vineyards to the interior parts.

#### 5.1.1. Alternative Host Plants

The presence of *Carpinus betulus* Linnaeus in woody vegetation bordering vineyards can serve as a potential source of the tetranychid mite *E. carpini* [139].

The thrips *Drepanothrips reuteri* (Uzel), *Thrips* spp. (e.g., *T. tabaci* Lindeman) and *Frankliniella occidentalis* (Pergande) (Thysanoptera: Thripidae) can damage grapevine shoots in early spring and summer, as well as berries from fruit set to harvest [117,140,141]. All these species are polyphagous and polyvoltine: *D. reuteri* on woody plants, while the others are mostly on herbaceous ones. Damage caused by *D. reuteri* occurs only in vineyards surrounded by woody vegetation with alternative hosts [81].

The Italian grape leafhopper *Zygina rhamni* Ferrari (Hemiptera: Cicadellidae) feeds on leaf mesophyll and chlorotic spots appear on the leaf blade [27]. It overwinters as adults and eggs on *Rubus* spp., on which it can complete a generation before migrating to vineyards [142,143]. This could suggest removing brambles from hedges and groves surrounding vineyards, even if this practice is challenging to implement and of uncertain effectiveness, since *Rubus* species also serve as overwintering sites for *A. atomus*, the main biological control agent of leafhoppers in vineyards [144,145,146].

The planthopper *Metcalfa pruinosa* (Say) (Hemiptera: Flatidae) is a very polyphagous phloem-feeding pest native to North America [147,148], that overwinters as eggs and completes one generation per year, with adults emerging from early July [149,150,151]. Infested plants are covered with waxy secretions and honeydew, on which sooty mold grows. Although *M. pruinosa* can develop on grapevines, large adult populations can colonize vineyards from surrounding hedgerows, where many host plants of this highly polyphagous planthopper are present [148,152]. This occurrence is more frequent when hedgerow plants are subjected to severe water stress that induces adults to migrate toward irrigated vineyards [152]. In the latter case, since removing hedgerows is not reasonable, extending irrigation to these may be an appropriate control strategy. After all, this appears to be the only viable option as the presence of honeybees collecting honeydew precludes the use of insecticides [67].

In summer, the presence of *D. vitifoliae* leaf galls at vineyard edges is sometimes associated with American vines in surrounding natural vegetation [153,154].

The fall webworm *Hyphantria cunea* (Drury) (Lepidoptera: Erebidae) is a moth native to North America whose gregarious larvae can sometimes defoliate completely deciduous trees [155], with a marked preference for *Morus alba* Linnaeus [156]. Occasionally, defoliation can be observed on grapevines [66,67]. Grapevines can be damaged by larvae hatched from eggs laid directly on grapevine leaves as well as larvae that, after completely defoliating nearby deciduous trees, move into contiguous vineyards to end their development. It is known that local food shortage triggers larval dispersal [157]. In any case, the presence of defoliated plants by the moth near the vineyards [67] should prompt careful sampling of the grapevines adjacent to these plants, possibly evaluating insecticide application at the vineyard edges.

The leafroller *Argyrotaenia ljungiana* (Thunberg) (Lepidoptera: Tortricidae) is a polyphagous and multivoltine moth that can cause damage to apple and pear orchards, eroding the fruits from the outside [158,159]. On grapevines, infestations were occasionally observed, especially during the second generation and in vineyards adjacent to apple orchards [43,160].

The poplar leaf roller *Byctiscus betulae* (Linnaeus) (Coleoptera: Rhynchitidae) is a univoltine and polyphagous pest that develops on many broadleaf plants (e.g., poplar, birch, chestnut) [161]. On grapevines, adults that have overwintered in various shelters (e.g., at the base of plants or under bark) colonize grapevines by eroding buds and young shoots and, after mating, females roll some leaves together to form a cigar-like structure, inside which they lay eggs and larvae develop [162,163]. Severe damage occurs only when many leaf rolls are present or when they incorporate grape clusters [43]. Infestations are typically located along the edges of hillside vineyards bordering woodlands, where alternative host plants are present [164].

The highly polyphagous and carpophagous *D. suzukii* infests grapevines primarily when other host plants, cultivated (e.g., cherry) or wild, are present in the surrounding landscape; in particular, unharvested infested fruits represent a source of adults for nearby vineyards [85].

#### 5.1.2. Host Plants of Pests, of Which the Grapevine Is Only a Feeding Host

The buffalo treehopper *Stictocephala bisonia* Kopp & Yonke (Hemiptera: Membracidae), native to North America, is a univoltine pest whose nymphs develop on various herbaceous plants [165]. However, the treehopper carries out part of its cycle on woody plants on which adults feed and females lay overwintering eggs. Adults feed radially on shoot axes of grapevines, causing annular strangling. Blockage of phloem flow causes carbohydrate accumulation in the distal part of the shoot, resulting in localized swelling just above the annular constriction, as well as leaf thickening and color change. Damage is more severe in young grapevines, as injured shoots cannot be used for the formation of trunk and cordons, with consequent delay of full yield. Such damage occurs if vineyards border meadows of grasses and legumes, on which nymphs can develop, and woody plants, on which females can lay overwintering eggs [165,166]. To prevent damage, in newly planted vineyards, the spontaneous herbaceous vegetation present in the inter-rows, mostly legumes, must be removed through soil tillage or managed with frequent mowing [43,167].

The European corn borer, *Ostrinia nubilalis* (Hübner) (Lepidoptera: Crambidae), is a polyphagous pest whose larvae bore holes mostly into corn plants, especially the panicle and stem, as well as into the stems and fruits of pepper and bean plants [64,168]. Since the 1980s, it has been reported as a carpophagous pest and shoot borer in fruit orchards (i.e., apple, peach and kiwi) [169,170,171], grapevine rootstock mother plants [172] and newly planted vineyards [173]. In grapevines, the injury consists of larval tunneling into the succulent portions of the current season’s shoots, causing either the death of tissues distal to the feeding site or predisposing canes to break, preventing their use for the formation of trunk and cordons. Damage during the grapevine rearing period has consistently been associated with the presence of nearby cornfields and with herbaceous plants with thick stems (such as species belonging to the genera *Amaranthus*, *Chenopodium*, *Artemisia*), growing along grapevine rows, on which egg masses and larval tunnels have been observed [170,173]. Nowadays, in northern Italy, although *O. nubilalis* is still reported as bivoltine [174], it develops three generations per year [175,176], due to climate warming [177]. In this context, second-generation females can be induced to migrate from already ripened cornfields towards surrounding areas and lay their eggs on herbaceous plants. If these are along the rows of newly planted vineyards, larvae can colonize the shoots and bore tunnels inside them. To prevent this damage, in vineyards located near cornfields, soil tillage to remove thick-stemmed herbaceous plants is recommended [173].

The risk of grapevine defoliation by *A. vitis* adults is more frequent when vineyards border permanent or multiannual meadows [67,75]. This risk increases further when the presence of meadows is concomitant with that of *Salix* spp. and *Tamarix gallica* Linnaeus, the preferred hosts of adults [44]. Under these conditions, when adults emerge, it is advisable to monitor the vineyard edges and, if necessary, apply an insecticide as soon as possible.

The Japanese beetle *Popillia japonica* Newman (Coleoptera: Scarabaeidae), native to eastern Asia, is a polyphagous and univoltine pest that has recently been introduced into Europe, where it is spreading [178]. Adults feed on broadleaf trees, including grapevines. Vineyard colonization is associated with the proximity to meadows, where larvae develop feeding on roots of herbaceous plants, and to wooded landscapes, where adults feed on broadleaved plants before returning to lay eggs in contiguous meadows [179].

The larvae of *Ametastegia glabrata* (Fallén) and *A. equiseti* (Costa) (Hymenoptera: Tenthredinidae) develop on *Rumex* sp. plants and overwinter in the pith of the branches, including renewal spurs of grapevines [180,181]. For this reason, the presence of these herbaceous plants along the rows of young grapevines must be avoided.

#### 5.1.3. Overwintering Host Plants for Adults of Pests

High population levels of first-generation *H. vitis* nymphs have been observed exclusively in vineyards contiguous to overwintering sites (i.e., plants with persistent leaves, even those planted for ornamental purposes) [26,27,29,182,183]. In particular, in the Friuli Venezia Giulia region (northeastern Italy), cases of heavy first-generation infestations reported over the past 40 years have consistently occurred in vineyards bordering parks densely planted with ornamental conifers.

The leafhopper *Erasmoneura vulnerata* (Fitch) (Hemiptera: Cicadellidae) was introduced to Europe from North America [184]. It overwinters as adults in rural buildings and hedgerows, and, in northeastern Italy, completes three generations per year [185]. Motile forms feed on leaf mesophyll and chlorotic spots are visible on the leaf blade. Economic damage can occur when feeding causes extensive leaf discoloration and premature leaf drop. Vineyard colonization by overwintered adults has shown an edge effect, suggesting the influence of nearby overwintering sites. Therefore, the proximity of rural buildings and hedgerows poses a risk to vineyards [185,186], so overwintering adults should be controlled at the vineyard edges to prevent summer damage.

The American grape leafminer *Phyllocnistis vitegenella* Clemens (Lepidoptera: Gracillariidae) was introduced into Europe from North America in the last decade of the 20th century [187]. The moth completes three generations per year and overwinters as adults in groves, especially with evergreen plants, so high infestation levels can occur in vineyard plots bordering these ecological structures [188]. For this reason, chemical control could be localized along vineyard edges.

#### 5.1.4. Summer Host Plants for Adults of Pests

The pale green plant bug *Apolygus spinolae* (Meyer-Dür) (Heteroptera: Miridae) is a univoltine true bug whose nymphs develop on grapevines [117,189] and sweet persimmon [190]. This species overwinters on grapevines as eggs in dormant buds, and in the spring, hatched nymphs feed on new shoots. It feeds on both still expanding leaves, causing deformation and perforation, and on young clusters, reducing fruit setting [189,191]. From late spring, adults migrate to natural vegetation, including herbaceous plants and woody species in hedgerows or groves, and return to grapevines in late autumn to lay overwintering eggs [192]. For this reason, infestations are more frequently observed in vineyards or their plots bordering natural vegetation [117,193].

### 5.2. Physical Exclusion of Pests

Insect-proof nets are the most used physical barrier between pests and plants [194]. Nets placed on the soil prevent the emerging *M. melolontha* adults from feeding on grapevine leaves [195].

As *D. suzukii* colonizes vineyards from outside, exclusion netting applied at veraison and removed at harvest time reduces larval infestation [196].

Banding the trunk with sticky barriers reduces the dispersion of *P. ficus* from the trunk to bunches [7]. Likewise, ants, which promote mealybug infestation by protecting them from natural enemies, are also hampered from colonizing grapevine parts infested by mealybug nymphs.

Since *T. ampellophaga* overwinters as young larvae in the pith of spurs or branches exposed by pruning cuts, sticky bands applied on the proximal part of fruiting arms hinder the migration of overwintering larvae to newly developing leaves [69].

Since noctuid larvae (*Noctua* spp.) emerge from the soil and feed at night on buds and new shoots, sticky bands, oilcloth strips or collars can be applied along the grapevine trunk to prevent their ascent and colonization of the grapevine [44,82,197]. The same sticky bands or fine mesh gauze can be used to avoid colonization and leaf erosion by the flightless adults of the weevil *Otiorhynchus sulcatus* (Fabricius) (Coleoptera: Curculionidae), which emerge from the soil after developing as larvae feeding on roots [67].

Some natural products can deter egg-laying and/or feeding activity. Kaolin hinders both the egg-laying and feeding activity of *H. vitis* [17,198] and reduces the population density of *E. vulnerata* [199]. Kaolin and sulfur dust showed an oviposition-deterrent effect on *L. botrana* [200,201]. In addition, kaolin hampers the feeding activity of *P. japonica* [202,203].

### 5.3. Diverting Pests from Grapevines

Colonization of grapevines by pests can be reduced by (i) retaining them on their herbaceous hosts, or (ii) attracting them to trap plants or artificial devices.

#### 5.3.1. Keeping Pests on Their Herbaceous Hosts

The presence of the thrips *T. tabaci* on grapevines is higher in vineyards with tilled and chemically weeded inter-rows [204], suggesting that herbaceous hosts’ removal favors the grapevine canopy colonization.

*Hyles livornica* (Esper) larvae (Lepidoptera: Sphingidae) develop on herbaceous plants, such as those belonging to the genera *Galium*, *Rumex*, and *Polygonum* [205], so the mowing of these plants growing along rows of young vineyards could favor grapevine colonization and defoliation.

Since herbaceous plants in vineyard inter-rows support noctuid larvae [44], early-season mowing could induce *Noctua* spp. larvae to colonise grapevines. For this reason, alternate mowing of inter-rows is suggested [67]. This practice represents a valid alternative to complete mowing of herbaceous vegetation in the inter-rows, also helping to preserve resources for natural enemies (i.e., prey, hosts, and alternative foods such as pollen and nectar) [206,207,208,209,210].

#### 5.3.2. Attracting Pests to Plant Traps or Artificial Devices

Mass trapping with yellow sticky traps is a common pest control strategy in greenhouses [211]. In vineyards, these traps, commonly used for the monitoring of leafhoppers and planthoppers [212,213] and for evaluating the effectiveness of insecticide applications [214,215], were recently proposed for the control of overwintering adults of *E. vulnerata* at vineyard edges [216].

*Lobesia botrana* and *E. ambiguella* overwinter under bark in silky cocoons [45,47]. Larvae can be induced to spin cocoons under pieces of cloth or inside bands of corrugated cardboard tied around woody parts of grapevines to simulate overwintering sites [44,217,218]. Cloth containing overwintering pupae should be removed in spring, just before the expected beginning of adult flight, to allow the pupal parasitoids to emerge and the predators overwintering under the bands to resume their activity.

The vine borer *Sinoxylon perforans* (Schrank) (Coleoptera: Bostrichidae) is a univoltine beetle that overwinters as adults and develops as a xylophagous insect on various broadleaf plants [66]. Adults overwinter on grapevines in galleries bored into canes during the summer, which compromises sap flow and, consequently, bunch development and ripening [67]. Moreover, these canes containing galleries cannot be used as fruiting arms, as they are prone to breaking during the pruning or in the subsequent vegetative season, and their buds fail to sprout [43,67]. In the spring, females lay eggs in galleries bored into dead wood, where the larvae develop, and adults emerge from early July. For this reason, after winter pruning, two-year-old arms must be removed from the vineyard as they serve as potential sites for larval development [67,219]. Moreover, bait fascines made of one- and more-year-old wood can be hung on grapevines to stimulate females to lay eggs on them; these fascines must be removed by the end of June, before adult emergence.

Mediterranean grape leaf beetle *Altica ampelophaga* Guérin-Méneville (Coleoptera: Chrysomelidae) is a monophagous and multivoltine leaf chewer that overwinters as adults and completes two to three generations per year [44,67]. Before grapevine leaf fall, artificial shelters, consisting of flat stones and plant residues, must be placed in the vineyards to attract overwintering adults and then removed before the adults begin to colonize the grapevines [44].

## 6. Direct Pests’ Control

Pests can be killed directly in their habitat by physical methods (e.g., mechanical tools and irrigation management) or after their collection.

### 6.1. Collection and Killing of Pests

Because *T. ampellophaga* overwinters as young larvae not only under grapevine bark but also inside the pith of two-year-old wood (i.e., spurs and fruiting arms of the previous year), removing pruning wood from vineyards before the larvae resume activity in early spring reduces the potential for infestation [64,67,68,69].

Grapevine bark hosting the pupal cocoons of *L. botrana* and *E. ambiguella* can be removed and burned or, better yet, placed in net containers with a mesh that retains the adults and allows the parasitoids to escape [218].

At egg hatching, grapevine leaves with aggregated *H. cunea* larvae can be manually removed when vineyard edges are infested [220].

After colonizing grapevines, adults of *A. ampelophaga* can be captured by *frappage* [44]. In spring, to prevent damage by the next generation, leaves infested by first-generation larvae, easily visible due to their gregarious behavior, can be removed [44,67,197].

At the beginning of infestation, the adults of *B. betulae* can be captured to reduce the number of cigar-like leaf rolls [44,67]. In particular, Della Beffa [221] suggests using *frappage* in the early morning, when adults are less mobile. Collecting cigar-like leaf rolls also helps reduce the infestation potential for the following year [44,197].

In spring, *frappage* can be used to capture wingless adults of *O. sulcatus* during the dark hours, because they remain mostly sheltered in the soil during daylight and colonize the vegetation after sunset [221].

*Vitisiella oenophila* (Haimhoffen) (Diptera: Cecidomyiidae) is a monophagous species on *Vitis*, overwintering in the soil as mature larvae and completing one to two generations per year [222]. Females lay eggs under the epidermis of young leaves and rachises. Larval activity induces the formation of lenticular galls between the two cuticles of mature leaves, whereas clusters appear twisted and young leaves are curled. It has been suggested that infested leaves be removed when larvae are already in the galls to reduce the potential infestation [67,197].

### 6.2. Killing Pests by Physical Methods

The grape rust mite *Calepitrimerus vitis* (Nalepa) (Acari: Eriophyidae) overwinters as a female under the outer bud scale and produces several generations per year [70,128]. On propagative planting materials, death of the entire buds can occur [223,224]. On young grapevines, feeding activity on growing leaves leads to the formation of chlorotic spots and deformations of the leaf blade. As a result, shoots exhibit shortened internodes, which can lead to poorer quality of canes, delaying the formation of the plant’s permanent structure. In summer, feeding activity on expanded leaves gives the upper surface a blackish rusty appearance. To have propagative planting materials free of *C. vitis*, hot-water treatment can be adopted [225].

Tilling the soil with rotary hoes causes pest death in their overwintering stage through mechanical destruction, deep burial and exposure to cold. This practice has been recommended against larvae of overwintering scarabaeids, *M. melolontha* and *P. bidens punctatus* [43,195], with an efficacy of 90% against the former species [195], as well as against larvae of *Noctua* spp. [67]. In autumn, soil tillage can also be adopted to bury the pupae of the last generation of *A. ampelophaga* before adult emergence [67], as well as the overwintering mature larvae of *V. oenophila* [222].

Bark stripping exposes *P. ficus* to mortality factors, including cool temperatures, natural enemies, and insecticides [7]. This practice, even without collecting the bark, still favors the mortality of overwintering pupae of grape berry moths, since it exposes them to cold temperatures and natural enemies [44,226].

The black grapevine scale *Targionia vitis* (Signoret) (Hemiptera: Diaspididae) is a polyphagous species that overwinters as fertile females, mostly hidden under the rhytidome, completing one or two generations per year [227]. Debarking and brushing the rhytidome are effective cultural practices.

Sprinkler irrigation reduces *P. ulmi* infestation compared to surface irrigation by physically removing mites [120]. Sprinkler irrigation, adopted in summer for thermoregulation, influences bunch colonization by *P. ficus* nymphs through mechanical disturbances and the creation of adverse microclimatic conditions, such as continuous leaf wetting and rapid temperature drops [228]. When *D. vitifoliae* was introduced into Europe, winter flooding of vineyards decreased phylloxera populations and damage [44,72]. Just as wind and rain cause mature larvae of *T. ampellophaga* to fall to the ground, preventing them from colonizing grapevines, spraying with water also causes the larvae to fall [65].

Moderate grapevine water stress (−1.0 MPa) during *H. vitis* egg-laying reduces leafhopper infestation [123,210] by increasing egg mortality [229]. Although water stress may negatively affect yield, qualitative benefits, particularly in red grape cultivars, can be expected [230,231,232,233], suggesting that irrigation should be applied only to avoid high or severe stress.

Bunch-zone leaf removal at the beginning of *L. botrana* egg-laying reduces larval infestation by around 50% [136,234], as it increases mortality of eggs and especially newly hatched larvae due to the very high temperatures reached by sun-exposed berries [235,236]. Although bunch-zone leaf removal can cause berry sunburn damage, combining it with kaolin not only reduces sunburn but also has a synergistic effect on *L. botrana* control [200,234]. The decrease in *L. botrana* infestation observed in table grapes cultivated under plastic cover [134,237] is likely due to the detrimental effect of high temperatures (>35 °C) on eggs and larvae.

Hot-water immersion treatment resulted in nearly 100% mortality of *P. ficus* on dormant grapevine cuttings used for nursery stock [238].

The larval nests of the first generation of grape berry moths are easily visible and can be crushed to destroy the larvae or pupae inside them [44,239]. Already over a century ago, this practice was considered highly effective in reducing the subsequent carpophagous generation and relatively inexpensive, as it often coincided with the removal of suckers.

## 7. Grapevine Yellows Diseases and Their Associated Vectors

The grape yellows diseases (GYDs), bois noir (BN) and flavescence dorée (FD), are two phytoplasmoses [240,241] that cause severe damage to European vineyards [242,243,244,245,246,247]. The phytoplasmas associated with these two GYDs are transmitted by insects belonging to the Hemiptera Auchenorrhyncha [248,249].

The phytoplasma associated with BN is the ‘*Candidatus* Phytoplasma solani’, belonging to the 16SrXII-A subgroup [250], that is transmitted from infected herbaceous plants to grapevines by the planthopper *Hyalesthes obsoletus* Signoret (Hemiptera: Cixiidae) [251], whose nymphs develop on roots of herbaceous hosts while adults can occasionally feed on grapevines [252]. Two main strains of the BN phytoplasma (i.e., tuf-type a and tuf-type b1), associated respectively with *Urtica dioica* Linnaeus and *Convolvulus arvensis* Linnaeus, were identified [253]. Herbaceous hosts of both the phytoplasma strains and *H. obsoletus* can be spread both inside (especially *C. arvensis*) and outside (especially *U. dioica*) vineyards [254,255,256].

The phytoplasma associated with FD belongs to the 16SrV taxonomic sub-group [241] and is transmitted from infected to healthy grapevines mostly by the leafhopper *Scaphoideus titanus* Ball (Hemiptera: Cicadellidae) [257,258,259]. *Scaphoideus titanus* is monophagous on *Vitis* spp. and a univoltine species introduced into Europe from North America [260,261]. It overwinters as eggs laid inside the bark of the woody parts of the grapevine, with nymphs hatching from mid-May and adults emerging from late June [259]. The spread of FD in vineyards is primarily caused by vectors becoming infected inside the vineyards after feeding on diseased grapevines. Consequently, roguing of symptomatic grapevines and chemical control of *S. titanus* are effective in preventing infections [262]. Control of FD is more difficult when infectious vectors colonize the vineyards from outside (American grapevines in hedges or groves, abandoned and cultivated vineyards that are untreated against the vector) [214,263,264].

### 7.1. Selection of Vineyard Planting Site

In grape-growing regions where *S. titanus* is absent, FD does not pose a problem, as alternative vectors of the phytoplasma, such as the planthopper *Dictyophara europaea* (Linnaeus) (Hemiptera: Dictyopharidae) [265] and the leafhopper *Orientus ishidae* (Mutsumura) (Hemiptera: Cicadellidae) [266], are not very efficient in the transmission of the FD phytoplasma [265,266,267,268,269,270,271]. Unfortunately, in Europe, the geographical distribution range of *S. titanus* is expanding northwards, due to climate warming, and southwards, likely due to the selection of populations adapted to warmer climates, resulting in the appearance of FD in previously unaffected areas [272]. As an example of expansion towards the north, in Switzerland, *S. titanus* was first found in southern Ticino in 1967 [273] and later in the North of the Alps in 1996 (i.e., Geneva) [274,275]; consequently, FD was first detected in Ticino (2004) and later in the North of the Alps (2005) [276]. As an example of southward expansion, in Italy, *S. titanus*, restricted to northern regions until the early 1990s [277], was reported in Tuscany (central Italy) in 1998 [278], followed by the first detection of FD in that region in 2003 [279]. Since the vector is expanding its distribution towards southern Italy [280,281] and central Europe [282], a further spread of FD towards the south and north is expected.

The nymphs of *H. obsoletus* develop in the soil, where they feed on the roots of herbaceous plants. The species prefers soils with a coarse pore matrix and low water retention capacity, as well as climatic regions with low average annual precipitation [283].

Vineyards planted in a habitat with infected American vines in hedgerows or groves may have a high incidence of FD, regardless of the insecticides applied against *S. titanus* [214].

### 7.2. Choosing the Grapevine Cultivar to Plant

Grapevine cultivars showed varying degrees of susceptibility (i.e., likelihood of showing symptoms) and sensitivity (i.e., severity of symptoms and ability to recover) to GYDs [243,246,284,285,286,287]. Therefore, in grape-growing regions affected by GYD epidemics, planting of the most susceptible and sensitive cultivars should be avoided until the disease prevalence returns to acceptable levels.

A decreasing gradient of infected grapevines from edges to the interior of vineyards occurs when the sources of phytoplasmas are external to vineyards (for BN: [255,288]; for FD: [214,264]. In such cases, the less susceptible cultivars must be planted along the edge rows of multi-cultivar vineyards to reduce the risk of disease spread.

### 7.3. Canopy Leaf-Density

*Scaphoideus titanus* prefers to inhabit the lower surface of the innermost leaves [289], which, in dense canopies, is difficult to cover with insecticides [290]. The effectiveness of contact insecticides is greater on suckers with well-exposed leaves than on basal leaves located within the canopy [19].

### 7.4. Interference with Vineyard and Grapevine Colonization by Vectors

#### 7.4.1. Removal of Plants’ External Sources of Infectious Vectors

The most effective and eco-friendly strategy to control the spread of GYDs is the removal of infected plants, which serve as sources of infectious vectors [291,292].

For BN, the removal of herbaceous plant hosts of both the phytoplasma and the vector can be obtained with tillage and chemical weeding [188,293,294,295,296,297,298,299]. However, since *H. obsoletus* adults prefer sparse vegetation on open soil [293,294] and their highest number in the grapevine canopy was observed in vineyards with bare soil [300], tillage may favor vineyard and grapevine colonization by the vector. In agreement with these data, tilled vineyards have a higher incidence of grapevines infected by BN phytoplasma than those with herbaceous vegetation [301]. Since *C. arvensis* colonizes bare soil [302], elective grassing and the use of cover crops have been suggested to reduce the soil surface available for *C. arvensis* spread and thereby suppress its growth [294,300,303]. For *U. dioica* control, frequent cutting of herbaceous vegetation along ditches and terraces bordering vineyards is an eco-friendly alternative to chemical weeding and tillage [304]. Since frequent mowing suppresses *U. dioica* through competition with other plants, its effect on stinging nettle reduction is not immediate, so in the first year of adoption, mowing should not be applied during the *H. obsoletus* flight period to not promote the colonization of the vineyard by adults [299]. Some studies have shown that other herbaceous plants, besides *U. dioica* and *C. arvensis*, can host ‘*Ca*. Phytoplasma solani’ and their removal should be considered if their role in the epidemiology of BN is demonstrated [292,305].

The removal of external grapevines that serve as sources of infectious *S. titanus* is the *sine qua non* for effective FD control, as insecticides applied in vineyards are otherwise insufficient to control FD [214]. Since, in the absence of American vine, the spatial distribution of symptomatic plants in vineyards is not affected by woody vegetation with broadleaf trees [306] and alternative FD vectors are less efficient in the transmission of FD phytoplasmas, the removal of other host plants (e.g., *Clematis vitalba* Linnaeus, *Alnus* spp., *Corylus avellana* Linnaeus, *Salix* spp.) [264,265,266,267,268,269,270] is not necessary, besides being difficult to implement due to their widespread occurrence.

#### 7.4.2. Physical Exclusion of Vectors

Nets 2.5 m high along vineyard edges have also been suggested as a method to prevent colonization by *S. titanus* adults [307,308] and it was also hypothesized for *H. obsoletus*.

#### 7.4.3. Diverting Vectors from Grapevines

In Israel, chaste tree (*Vitex agnus-castus* Linnaeus) is the preferred host for *H. obsoletus* [309,310], and a “push and pull” strategy, based on the use of chaste trees as a trap plant at vineyard edges, has been suggested to divert the vector away from vineyards [310]. However, this strategy is not applicable in Europe because *V. agnus-castus* has been found to host the BN phytoplasma [311,312].

To reduce the probability that *H. obsoletus* adults colonize the grapevine canopy, the removal of suckers along the trunk and canopy shoots closer to the ground before adult emergence is highly recommended [313].

In May–June, nymphs of *S. titanus* are mainly aggregated on suckers growing along grapevine trunks [260,289,314,315,316]. This occurs because many nymphs hatch from eggs laid on the trunk [317] and nymphs that fall to the ground when the grapevine is shaken recolonize grapevines starting from suckers [315]. The removal of these shoots can reduce the nymph survival because nymphs that fall to the ground have more difficulty colonizing grapevine leaves [318,319]. This also explains why sucker removal is more effective in horizontal than vertical training systems, as the greater distance between ground level and the nearest canopy shoots makes it even more difficult for nymphs to colonize the plant.

### 7.5. Direct Vector Control

Tillage practices, such as weeding in late summer and plowing in winter, effectively control BN by removing herbaceous plants that serve as sources of the phytoplasma and by causing mortality of *H. obsoletus* nymphs both mechanically and through exposure to winter cold [293,294]. The strong sensitivity of planthopper populations to low temperatures has been linked to their collapse in years with deep frosts that occur after the overwintering nymphs have already risen to the surface [256,288].

Overwintering eggs of *S. titanus* are primarily laid under the bark of permanent wood and fruiting arms from the previous growing season, which are removed during winter pruning [260,261,319,320]. The destruction of pruned arms reduces nymph populations [318,319]. Since *S. titanus* can occasionally lay eggs on one-year-old wood [260,261] used as grapevine propagative material [317,321], hot-water treatment of rootstocks and scions is suggested even if its efficacy is not 100% [321].

## 8. Cultural Control vs. Conservation Biological Control

The removal of ecological infrastructures (e.g., groves, hedgerows, permanent green cover), which can serve as potential sources of pests and vectors, is a control strategy that is not always practicable and sometimes may even be adverse, as it conflicts with the principle of conservation biological control based on plant and habitat diversification [322,323,324,325].

The role of vegetational diversity in controlling vineyard pests is ambiguous, as it can be a source not only of pests but also of their natural enemies [326]. Therefore, the removal of ecological infrastructure could favor the control of some pests and disadvantage the control of others by reducing the presence and activity of their natural enemies. Ideally, each case should be assessed individually, with plant diversity managed by carefully selecting which species should or should not be present in the inter-rows and surrounding vegetation of the vineyards [327,328].

## 9. Final Consideration

When chemical control was unavailable, decisions regarding vineyard design and annual agronomic practices also took into account the effects on pests. This clearly emerges from old handbooks on grapevine pests, which dedicated large sections to describing these aspects. Later, all crop choices were aimed at production, as insecticides were available for pest control. Today, these practices are becoming relevant again. Farmers are called upon to avoid practices that favor pests while also adopting specific ones to help control them.

Nowadays, research, although oriented towards highly technological but not yet immediately applicable tools, will have to again turn its attention to the development of cultural control strategies, many of which also interfere with conservative biological control based on habitat management.

## Figures and Tables

**Table 1:** Outline of the pest control strategies related to the present review. The numbering in the table refers (i) to headings and (ii) subheadings reported in the text and in Table 2.

Headings	Subheadings
3. Selection of the vineyard planting site	3.1. Climatic conditions
	3.2. Soil characteristics
	3.3. Other habitat components
4. Host-plant-mediated pest control	4.1. Choosing *Vitis* species or cultivar to plant
	4.2. How to grow grapevines
	4.2.1. Fertilization and irrigation
	4.2.2. Training systems and pruning
	4.2.3. Harvest time
5. Interference with vineyard and grapevine colonization by pests	5.1. Removal of pests’ source plants
	5.1.1. Alternative host plants
	5.1.2. Host plants of pests, of which the grapevine is only a feeding host
	5.1.3. Overwintering host plants for adults of pests
	5.1.4. Summer host plants for adults of pests
	5.2. Physical exclusion of pests
	5.3. Diverting pests from grapevines
	5.3.1. Keeping pests on their herbaceous hosts
	5.3.2. Attracting pests to plant traps or artificial devices
6. Direct pests’ control	6.1. Collection and killing of pests
	6.2. Killing pests by physical methods
7. Grapevine yellows diseases and their associated vectors	7.1. Selection of vineyard planting site
	7.2. Choosing the grapevine cultivar to plant
	7.3. Canopy leaf-density
	7.4. Interference with vineyard and grapevine colonization by vectors
	7.4.1. Removal of plants’ external sources of infectious vectors
	7.4.2. Physical exclusion of vectors
	7.4.3. Diverting vectors from grapevine
	7.5. Direct vector control

**Table 2:** List of the grapevine pests. Mites are grouped by family and insects by order. Only for Hemiptera, the suborder and section are considered. Within each group, the species are listed in alphabetical order. (X) indicates the subheading in which each arthropod pest is considered.

Arthropod Pest	Control Strategy (N. of Subheading)															
	3.1.	3.2.	3.3.	4.1.	4.2.1.	4.2.2.	4.2.3.	5.1.1.	5.1.2.	5.1.3.	5.1.4.	5.2.	5.3.1.	5.3.2.	6.1.	6.2.
Tetranychidae																
*Eotetranychus carpini*	X			X				X								
*Panonychus ulmi*					X											X
Eriophyidae																
*Calepitrimerus vitis*																X
*Colomerus vitis*						X										
Thysanoptera																
*Drepanothrips reuteri*								X								
*Thrips* spp.								X					X			
*Frankliniella occidentalis*								X								
Hemiptera Heteroptera																
*Apolygus spinolae*											X					
Hemiptera Homoptera Auchenorrhyncha																
*Erasmoneura vulnerata*										X		X		X		
*Hebata vitis*	X			X	X	X				X		X				X
*Jacobiasca lybica*	X															
*Metcalfa pruinosa*								X								
*Stictocephala bisonia*									X							
*Zygina rhamni*								X								
Hemiptera Homoptera Sternorrhyncha																
*Daktulosphaira vitifoliae*		X		X	X			X								X
*Parthenolecanium corni*	X				X											
*Planococcus ficus*				X	X	X	X					X				X
*Targionia vitis*																X
Lepidoptera																
*Argyrotaenia ljungiana*								X								
*Cryptoblabes gnidiella*			X	X			X									
*Eupoecilia ambiguella*	X		X	X			X							X	X	
*Hyles livornica*													X			
*Hyphantria cunea*								X							X	
*Lobesia botrana*	X		X	X	X	X	X					X		X	X	X
*Noctua* spp.			X	X									X			
*Ostrinia nubilalis*	X *								X							
*Phyllocnistis vitegenella*										X						
*Theresimima ampellophaga*	X											X			X	X
Coleoptera																
*Altica ampelophaga*														X	X	X
*Anomala vitis*		X							X							
*Byctiscus betulae*								X							X	
*Melolontha melolontha*		X										X				X
*Otiorhynchus sulcatus*												X			X	
*Pentodon bidens punctatus*		X			X											X
*Popillia japonica*									X			X				
*Sinoxylon perforans*														X		
Diptera																
*Drosophila suzukii*			X	X				X				X				
*Vitisiella oenophila*															X	X
Hymenoptera																
*Ametastegia* spp.									X							

* Information discussed in Section 5.1.2.

## Data Availability

No new data were created or analyzed in this study. Data sharing is not applicable to this article.

## Supplementary Materials

The following supporting information can be downloaded at: https://www.mdpi.com/article/10.3390/insects17010113/s1, Table S1: Biology and damage caused by grapevine pests and vectors considered in this review.

## Abbreviations

The following abbreviations are used in this manuscript:

BN	bois noir
FD	flavescence dorée
GYDs	grape yellows diseases
GLD	grapevine leafroll disease
GLRaV-3	grapevine leafroll-associated virus 3
GPGV	grapevine Pinot gris virus
IPM	Integrated pest management
